# Supported cognitive-behavioural self-help versus treatment-as-usual for depressed informal carers of stroke survivors (CEDArS): study protocol for a feasibility randomized controlled trial

**DOI:** 10.1186/1745-6215-15-157

**Published:** 2014-05-06

**Authors:** Joanne Woodford, Paul Farrand, Edward R Watkins, David A Richards, David J Llewellyn

**Affiliations:** 1 Mood Disorders Centre, Psychology, College of Life and Environmental Sciences, University of Exeter, Perry Road, Exeter EX4 4QG, UK; 2 Institute of Health Research, University of Exeter Medical School, Heavitree Road, Exeter EX1 2 LU, UK; 3 Epidemiology and Public Health Group, University of Exeter Medical School, Heavitree Road, Exeter EX1 2 LU, UK

**Keywords:** Randomized controlled trial, Cognitive behavioral therapy (CBT), Self-help, Depression, Stroke, Informal caregivers

## Abstract

**Background:**

Increased life expectancy has resulted in a greater provision of informal care within the community for patients with chronic physical health conditions. Informal carers are at greater risk of poor mental health, with one in three informal carers of stroke survivors experiencing depression. However, currently no psychological treatments tailored to the unique needs of depressed informal carers of stroke survivors exist. Furthermore, informal carers of stroke survivors experience a number of barriers to attending traditional face-to-face psychological services, such as lack of time and the demands of the caring role. The increased flexibility associated with supported cognitive behavioral therapy self-help (CBTsh), such as the ability for support to be provided by telephone, email, or face-to-face, alongside shorter support sessions, may help overcome such barriers to access. CBTsh, tailored to depressed informal carers of stroke survivors may represent an effective and acceptable solution.

**Methods/Design:**

This study is a Phase II (feasibility) randomized controlled trial (RCT) following guidance in the MRC Complex Interventions Research Methods Framework. We will randomize a sample of depressed informal carers of stroke survivors to receive CBT self-help supported by mental health paraprofessionals, or treatment-as-usual. Consistent with the objectives of assessing the feasibility of trial design and procedures for a potential larger scale trial we will measure the following outcomes: a) feasibility of patient recruitment (recruitment and refusal rates); (b) feasibility and acceptability of data collection procedures; (c) levels of attrition; (d) likely intervention effect size; (e) variability in number, length and frequency of support sessions estimated to bring about recovery; and (f) acceptability of the intervention. Additionally, we will collect data on the diagnosis of depression, symptoms of depression and anxiety, functional impairment, carer burden, quality of life, and stroke survivor mobility skill, self-care and functional ability, measured at four and six months post-randomization.

**Discussion:**

This study will provide important information for the feasibility and design of a Phase III (effectiveness) trial in the future. If the intervention is identified to be feasible, effective, and acceptable, a written CBTsh intervention for informal carers of stroke survivors, supported by mental health paraprofessionals, could represent a cost-effective model of care.

**Trial registration:**

Current Controlled Trials ISRCTN63590486.

## Background

Technological advances in healthcare have resulted in increased life expectancy across the developed world [1]. However, such increases have led to significant challenges, with excessive demand placed upon the provision of treatment and care of patients with chronic physical health conditions [1,2]. This has resulted in an increased reliance on informal care within the community for people with chronic physical health conditions [3]. However, increased provision of informal care places informal carers at greater risk of poor mental and physical health [4-6] accompanied by reduced opportunity for paid employment and social activity [7]. On average, 30% of informal carers experience depression [8]. Rates are even higher when the chronic physical health condition causes significant behavioral, cognitive, and emotional impairment in the patient being cared for [9]. Significant forms of impairment are experienced with stroke [10], with 30 to 68% of informal carers of stroke survivors experiencing depression from the time of the initial stroke to three years post-stroke [11,12]. Given such a high prevalence of depression, demand for accessible evidence-based psychological interventions targeted at informal carers of stroke survivors is high.

Although evidence-based psychological treatments for depression exist [13] the costs of service delivery are high, with demand for treatment exceeding the capacity of therapists, resulting in long waiting lists [14] and limited access [15]. Additionally, informal carers experience specific barriers to accessing primary care services [16]. Barriers have included a lack of recognition of the difficulties associated with the caring role by health professionals [16], with general practitioners more likely to provide practical support rather than referral for formal psychological treatment. Additionally, interventions are predominantly focused on the stroke survivor rather than the informal carer [17,18]. The longterm psychological needs of informal carers of stroke survivors have therefore been largely neglected, making it difficult for informal carers to access evidence-based psychological therapies [19-21].

To improve access to evidence-based psychological therapies, there have been movements towards the use of supported cognitive behavioral therapy self-help (CBTsh) for the treatment of mild to moderate depression and anxiety disorders [22]. Meta-analyses of supported CBTsh provide evidence that it is an efficacious treatment for depression and anxiety [23-26]. Furthermore, when compared with traditional CBT no significant difference in overall effect size was found, suggesting supported CBTsh and traditional CBT are comparable treatments for both depression and anxiety [27]. CBTsh is not delivered by a therapist, rather CBT specific principles are communicated to the patient through the use of self-help materials, commonly in a written or internet-based format [22]. Guidance and motivation appear to increase effectiveness [24] although the need for support differs across mental health conditions [25]. To further increase access, support is provided in a variety of ways including telephone, email, or face-to-face [22]. Because the demands of caring are often a barrier to attending therapy [16,28], the increased flexibility associated with the delivery of CBTsh may increase access to appropriate psychological support for informal carers of stroke patients.

Although CBTsh interventions are available for depression, evidence highlights that significant adaptations to interventions may be required prior to application to different depressed populations, for example, to those with depression co-morbid to a physical health condition [29]. Additionally, although CBTsh appears effective for common mental health difficulties, reviews of CBTsh interventions for people with physical health conditions are less promising [30-32]. This raises the possibility that benefits demonstrated in general CBTsh interventions for adult depression may not generalize to medical populations that have depression as a secondary comorbidity or informal carers of people with physical health conditions. Indeed, mental health services for carers have been criticized for not being tailored to address the unique difficulties informal carers experience [33]. Such difficulties include informal carers managing behavioral problems [34], physical impairments [34] and cognitive impairment [35], all of which are experienced by informal carers of stroke survivors [9]. To the best of our knowledge, only three published studies have examined CBTsh for depression within informal carer populations, specifically carers of people with anorexia nervosa [36,37] and cancer patients [38]. It is therefore clear that more research is required into both the effectiveness and acceptability of CBTsh interventions for the treatment of depression in informal carers of patients with chronic physical health conditions.

Over the last decade there has been a growing recognition of the importance of understanding patients’ experiences when developing health resources [39,40] and healthcare policy [41]. Reflecting this recommendation, a new written CBTsh self-help intervention has been developed specifically targeted at depressed informal carers of stroke survivors [42]. The content was informed through a series of qualitative studies to understand the specific difficulties and challenges experienced by depressed informal carers of stroke survivors, and helpful coping strategies used by currently non-depressed informal carers. The new CBTsh intervention recognizes and targets the difficulties commonly experienced by informal carers of stroke survivors identified through the qualitative studies. Additionally, helpful coping strategies used by currently non-depressed informal carers were used to further inform and adapt the content of written CBTsh intervention. Recognizing such strategies may provide a useful aid to informal carers experiencing emotional difficulties [43]. This study seeks to examine the feasibility of running a definitive randomized controlled trial (RCT) to examine the effectiveness acceptability of this specially adapted CBTsh intervention.

### Study aims and objectives

We will conduct a feasibility phase II RCT [44,45] comparing a written CBTsh intervention for depressed informal carers of stroke survivors supported by paraprofessional mental health workers (Psychological Wellbeing Practitioners; PWPs) with treatment-as-usual (TAU). Outcomes will assess a number of methodological and procedural uncertainties that require investigation prior to designing and applying for funding for a Phase III trial. Therefore the following four questions will be addressedFor informal carers of stroke survivors receiving CBTsh in a fully powered phase III trial, what would be the estimates of likely recruitment and retention rate; estimates of the range of effect sizes; feasibility and acceptability of data collection methods and instruments; and acceptability and structure of the treatment procedures to participants?

## Methods/Design

### Study design

We will conduct a single blind parallel group feasibility RCT comparing CBTsh for depressed informal carers of stroke survivors (intervention group) with TAU (control group). This protocol follows CONSORT [46] and SPIRIT [47] guidelines for reporting clinical trial protocols.

### Setting

We will recruit participants over a six-month period through primary care services, specialist stroke healthcare settings, and community organizations in the counties of Cornwall and Dorset (southwest England). Participants will be treated within primary care mental health services commissioned under the Improving Access to Psychological Therapies program (IAPT) [48].

### Participant inclusion criteria

Eligible participants will be self-identified informal carers of stroke survivors at a minimum of two months post-home discharge (relating to the time of the most recent stroke), aged 16 and over. We will recruit participants meeting the 10th revision of the International Statistical Classification of Diseases and Related Health Problems (ICD-10) criteria for major depression as determined by the Clinical Interview Schedule (CIS-R) [49] and who score between 10 and 22 on the Patient Health Questionnaire-9 (PHQ-9) [50]. To reflect standard practice participants will be eligible to participate in the study whether or not they are currently receiving antidepressant medication, however the dose must have been stable for at least one month prior to recruitment into the study. All participants need to be able to read in English in order to engage with the written CBTsh intervention.

### Participant exclusion criteria

Potential participants with post-traumatic stress disorder (PTSD), psychosis, bipolar disorder, current substance or alcohol abuse, or who are acutely suicidal will be excluded from participation in the study, in addition to those currently receiving formal psychotherapy for their depression.

### Recruitment settings and procedure

A number of recruitment techniques will be utilized including letter mail-out, the use of brochures, posters and flyers, advertisement in newsletters, and direct referral from healthcare professionals. Such multifaceted recruitment techniques have been successfully used to recruit informal carers of people with dementia [51]. Details of the recruitment strategies to be used within each recruitment setting are detailed below.

### Primary care

Participants will be recruited by searching GP records, a strategy successfully employed within other depression trials [52-54]. First, practice staff will search general practice electronic case records for stroke survivors. Practice staff will subsequently manually screen these records to identify stroke survivors who have a known informal carer. Practice staff will send a study invitation pack to all identified informal carers inviting them to take part, including an invitation letter, patient information sheet, and reply slip. Informal carers will reply directly to the research team to express whether they would like to be contacted to discuss the research in more detail either by using the reply slip or calling the research team directly. Additionally, GPs will be able to directly refer suitable informal carers to the study team and study posters will be displayed in practice reception rooms to further advertise the study.

### Specialist stroke care settings

We will also recruit participants from clinical acute and community based stroke healthcare settings, for example acute stroke units, stroke rehabilitation units, community early discharge, and rehabilitation teams. Stroke research nurses and community stroke healthcare professionals will approach informal carers seen within these settings and provide brief details about the study and a study invitation pack. If interested, informal carers can either consent for their contact details to be sent to the research team or reply directly to the research team themselves.

### Community outreach

Participants will also be recruited through a variety of community based stroke and informal carer charities such as the Stroke Association, Different Strokes, and community stroke clubs and groups. Groups and charities interested in supporting the study will be provided with brochures and flyers advertising the study to hand out to informal carers. The research team will also endeavor to give presentations to members of stroke and informal carer groups to further advertise the research program. Additionally, the study will be advertised in stroke and informal carer charity newsletters.

### Reasons for non-participation

All study invitation packs will also include anonymized reply slips with space for writing reasons for non-participation and researchers will ask participants for reasons from those who verbally decline. It will be made clear that researchers will not be trying to persuade participants to reconsider their decision. This information will provide further information in terms of the feasibility of recruitment and acceptability of the intervention.

### Screening, baseline and informed consent

**Figure 1:**
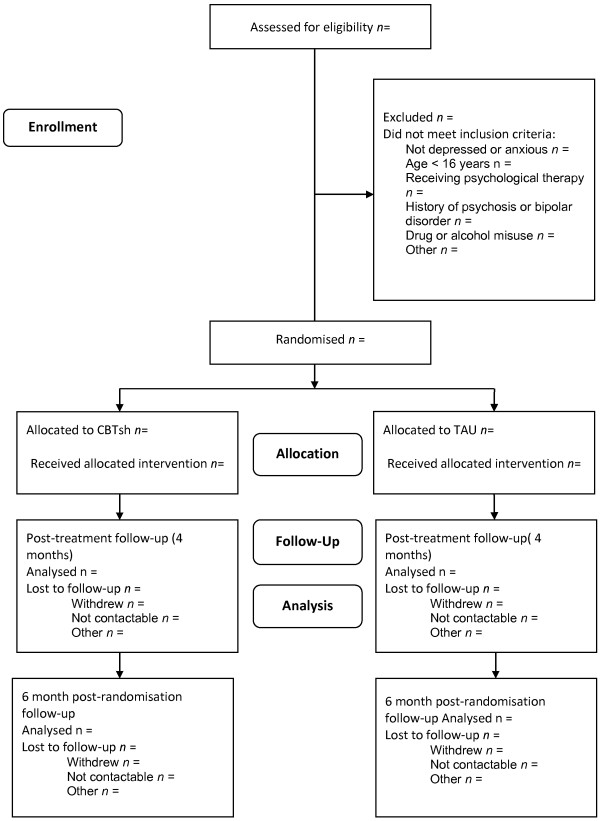
CONSORT diagram.

A researcher will speak to all informal carers of stroke survivors about the study in more detail. If interested in participating in the study, informal carers will be asked to provide verbal consent for a telephone screen to be conducted against the inclusion criteria to confirm the current level of depressive symptoms, length of time caring, and any history of PTSD, psychosis, bipolar disorder, and current substance or alcohol abuse. If eligible to participate, dependent upon preference, the potential participant will be invited to attend a full screening appointment via the telephone or face-to-face, to confirm a diagnosis of major depression using the CIS-R. If eligible, the full baseline assessment will be undertaken. Potential participants will be required to provide full written informed consent before the full screening appointment or baseline can take place. Once the full baseline assessment has taken place, participants will be randomized (see Figure 1).

### Randomization and allocation concealment

We will randomly allocate eligible participants to one of the two study arms - supported CBTsh or TAU - using a web-based randomization service at the Peninsula Clinical Trials Unit which will be concealed from the research team. We will use minimization to ensure balance between arms in relation to site in order to assist with efficient study management (East Dorset, West Dorset, North Cornwall, and South Cornwall) and factors that may affect the outcomes: PHQ-9 score (Moderate: PHQ-9 score 10 to 14; Moderately Severe: PHQ-9 score 15 to 22) and sex (male or female). The minimization algorithm will contain a stochastic element to maintain a degree of unpredictability when allocating. In order to preserve the blinding of research personnel participants will be informed as to which study arm they have been allocated by a researcher not otherwise associated with the study.

### Sample size

No formal power calculations are usually undertaken in feasibility RCTs [55]. Instead a sufficient sample size to calculate the critical parameters relating to the feasibility outcomes in the trial, for example recruitment and attrition rates [55], should be used. As such, we will use the recommended sample size of 30 participants per arm for feasibility studies [56] consistent with the median sample size found in both feasibility and pilot RCTs [57]. This will provide a reasonable indication of the likely sample size required for a larger trial [45,58].

### Treatments

#### Intervention

Participants will receive one assessment session and up to twelve support sessions. Limiting the number of sessions used to support the intervention is identified as one of the characteristics associated with low-intensity CBT [59]. The number of support sessions received will be decided collaboratively between the PWP and participant. The initial assessment session will be of 35 minutes duration, with subsequent sessions lasting between 25 and 35 minutes each. During their first assessment session participants will receive the CBTsh introduction workbook specifically developed for the trial [42]. The assessment session will mainly comprise a client-centered assessment to understand the difficulties experienced by the participant alongside the provision of information concerning the impact of depression, the impact of the caring role on mood, and the CBT approach. Participants will also be provided with more information about each of the three possible interventions (behavioral activation, problem solving, and goal setting) by the PWP, supplemented by the introduction workbook [42]. During the first support session participants will be provided with an additional workbook detailing the particular CBTsh intervention they wish to use (behavioral activation, problem solving, or goal setting). Subsequent support sessions will provide guidance and encouragement around the use of the chosen CBTsh intervention. All assessment and support sessions will follow a structured treatment support protocol [60] adapted to fit the needs of carers that were identified within the treatment development phase. Assessment and support sessions will be provided either face-to-face, over the phone, or through a combination of both methods as determined by participants’ preference. All participants will receive relapse prevention during their final support session, again guided by a relapse prevention workbook developed for the study.

Those providing support will be qualified PWPs trained in accordance with the curriculum supporting the Improving Access to Psychological Therapies Programme [61,62] to deliver low-intensity CBT-based interventions for depression and anxiety [63]. PWPs will also receive an additional one day training session delivered by the first author, alongside a carer of a stroke survivor and a stroke healthcare professional. Consistent with IAPT supervision guidance for the PWP workforce [64] PWPs will be provided with weekly case management supervision to provide advice and support by an experienced mental health professional within the service. Case management supervision will be delivered to predetermined protocols ensuring all participants are brought to supervision at preset times during treatment or when they display particular clinical characteristics and risk [63]. PWPs supporting the intervention will also be provided with group clinical supervision by an IAPT clinical educator once a month. This supervision will focus on the discussion of cases and ongoing clinical skills development, will last approximately 45 minutes, and be provided for the duration of the treatment phase of the trial.

### Control - treatment-as-usual

Participants randomized to the control condition will receive usual care delivered by their general practitioner or other healthcare provider. In general, this may include a consultation with their general practitioner, the prescription of antidepressant medication, or a referral to a mental health service for psychological intervention.

### Blinding

The study is single blind, with the research staff conducting outcome assessment interviews remaining blind to group allocation. Participants will be reminded not to disclose the arm they have been randomized to during contact with the researcher throughout the duration of the trial. To maintain blinding of the outcome assessor, acceptability interviews will be conducted by a researcher not otherwise associated with the study. Due to the nature of the intervention, participant and clinician blinding is not possible.

### Outcome measurements

#### Feasibility outcome measurements

The feasibility of participant recruitment will be examined including numbers assessed for eligibility; numbers eligible; reasons for ineligibility; reasons for non-participation and numbers randomized. Additionally comparisons will be made between recruitment settings and recruitment techniques.

The relative levels of diagnosis of depression between treatment arms at post-treatment (four months) will be determined using the CIS-R.

The feasibility and acceptability of data collection processes will be investigated through the number of missing items and follow -up rates relating to the clinical outcome measurements likely to be used in a Phase III trial. Additionally, we will examine levels of attrition through treatment and study drop-out rates.

The range of number, length, and frequency of support sessions required to bring about recovery from depression, defined as a score of ≤9 on the PHQ-9, as per current IAPT guidance [65] will also be reported.

The acceptability of the treatment will be examined through reasons reported for not attending support sessions, reasons for withdrawal from treatment and acceptability interviews will be conducted at four months post-randomization.

PWP adherence to the protocol will be examined using audio tapes of treatment sessions. Levels of PWP adherence to the protocol will be judged by a member of academic staff involved in delivering PWP training on an accredited PWP training programme. A randomly selected sample of 20% recorded treatment sessions for each PWP in the study will be assessed for levels of adherence.

### Clinical outcome measurements

A number of clinical outcome measurements have been included to examine the feasibility of the proposed data collection process, estimates of relative levels of diagnosis of depression between arms at post-treatment and the range of number, length, and frequency of support sessions required to bring about recovery from depression. The CIS-R will be used to assess diagnosis of depression and the PHQ-9 will be taken to measure depression severity. Symptoms of anxiety will be measured using the Generalized Anxiety Disorder 7-item Scale (GAD-7) [66] and levels of functional impairment will be measured using the Work and Social Adjustment Scale (WSAS] [67]. Stroke survivors’ mobility skill and self-care will be examined with the Barthel Activities of Daily Living Index (BI) [68]. The measure will be completed by the informal carer on behalf of the stroke survivor. Discrepancies arise between stroke survivor and informal carer assessment of stroke survivor functional ability, with carers rating patients as more disabled [69]. However, such disagreement has been found to be associated with increased carer burden [69] and is therefore of interest to collect. Stroke survivors’ level of functional impairment will be measured through the Frenchay Activities Index (FAI) [70]. The measure will be completed by the informal carer on behalf of the stroke survivor. Due to bias found when using proxy scores on the FAI [71] results will be interpreted with caution. However the use of proxy measurements on the FAI are considered suitable for research purposes [72]. Carer burden will be measured using the Caregiver Burden Scale (CBS) [73]. We will also measure informal carer quality of life using both the Short Form (36) Health Survey (SF-36) [74]; and the EuroQol-5D (EQ-5D) [75]. Finally, health and public service use will be collected using an adapted version of the Client Socio-Demographic and Service Receipt Inventory (CSRI) [76]. The version of the CSRI for use in this study has been adapted from the original CSRI [76] and a further version developed for informal carers of stroke survivors [77].

### Demographics

Several background and socio-demographic variables will be collected at screening for informal carers and stroke survivors.

The collected variables for an informal carer: source of referral; age; gender; ethnic background; relationship status; relationship to stroke survivor; employment status; yearly household outcome; highest level of academic qualification; length of time caring; whether lives with the stroke survivor; provision of care before the stroke; receipt of support services in the home; hours of support services received in the home per week, and hours of caring per week.

The collected variables for a stroke survivor: age; gender; ethnic background; relationship status; employment status; date of first stroke; date of most recent stroke; type of first stroke (ischemic, hemorrhage, transient ischemic attack (TIA)); type of most recent stroke, (ischemic, hemorrhage, TIA) and whether the stroke survivor is aphasic.

### Data collection

Dependent upon participant preference, the researcher will collect data either over the telephone or face-to-face at screening, baseline, four months and six months post-randomization. The adapted CSRI will be collected via post due to the potential for unblinding as the measure includes information about the receipt of psychological treatment. A summary of outcomes collected at each time point can be seen in Table 1.

**Table 1:** Study clinical outcome measures by time point

**Outcome measure**	**Time point**
Demographics (informal carer)	Initial screen
Demographics (stroke survivor)	Initial screen
PHQ-9	Initial screen; four months post-randomization; six months post-randomization
GAD-7	Initial screen; four months post-randomization; six months post-randomization
CIS-R	Full screen; four months post-randomization; six months post-randomization
WASAS	Baseline; four months post-randomization; six months post-randomization
CBS	Baseline; four months post-randomization; six months post-randomization
BI	Baseline; four months post-randomization; six months post-randomization
FAI	Baseline; four months post-randomization; six months post-randomization
SF-36	Baseline; four months post-randomization; six months post-randomization
EQ-5D	Baseline; four months post-randomization; six months post-randomization
Health and public service use	Baseline; four months post-randomization; six months post-randomization

*BI*, Barthel activities of daily living index; *CBS*, Caregiver burden scale; *CIS-R*, Clinical interview schedule; *EQ-5D*, EuroQol-5D; *FAI*, Frenchay activities index; *GAD-7*, Generalized anxiety disorder 7-item scale; *PHQ-9*, Patient health questionnaire-9; *SF-36*, Short form (36) health survey; *WSAS*, Work and social adjustment scale.

### Acceptability of the intervention

#### Study objectives and design

We will conduct a sub study to examine the following question: what are participants’ views on the acceptability of CBTsh? Semi-structured interviews will be conducted with all participants randomized to receive CBTsh to examine the acceptability of the new intervention. Open-ended questions will be asked around participants’ impressions of supported CBTsh; the relevance and suitability of the intervention for carers and relatives of stroke survivors; receiving support; specific interventions used; perceived benefit and impact of the intervention; difficulties experienced using the intervention; continued use of self-help strategies; and recommendations for future development. The topic guide has been partially informed by a previous qualitative study investigating the acceptability of online-based CBTsh for depressed patients with multiple sclerosis [29]. Non-attendees and poor attendees will also be asked about reasons for dropping out of the intervention and to consider what a more acceptable intervention may look like. Interviews will be semi-structured and conducted over the telephone. Interviews are anticipated to last between 45 and 90 minutes, however the duration may be shorter for those categorized as non-attendees and poor attendees.

### Sampling

All participants allocated to receive the intervention will be invited to participate. Dependent upon attendance of support sessions, participants will be categorized into one of the following: (i) non attendees, defined as not attending any sessions; (ii) poor attendees, defined as attending the assessment session and then terminating treatment before reaching a shared decision with the PWP to be discharged from treatment; or (iii) completers, defined as those who engage in treatment until a shared decision is made with the PWP to terminate treatment.

### Statistical analysis

#### Quantitative

Data analysis will mainly be descriptive and address the primary outcomes relating to the feasibility of conducting a future definite RCT. Participant flow will be summarized following the CONSORT diagram [46]. Recruitment and attrition rates (both treatment and study dropouts) will be calculated, along with 95% confidence intervals. Protocol deviations, along with reasons and number of missing items on questionnaires will be reported. The mean and standard deviation for each outcome measurement will be reported at baseline, four, and six months. The mean and standard deviation will also be reported for the number, length, and frequency of support sessions required to bring about recovery.

### Health economics

Estimates of cost-effectiveness will not be possible due to the design reflecting a feasibility RCT. However the feasibility and acceptability of collecting outcome measurement relating to health-related quality of life and patient NHS and social support use will be examined. Processes for estimating costs of delivering the intervention will also be tested. The Short Form-6 dimension (SF-6D) [78] will be used to gain measures of utility from 11 items of the SF-36 covering 6 dimensions (physical functioning, social functioning, role limitations, mental health, vitality, and pain). Both the SF-6D and EQ-5D will be used to determine quality-adjusted life years (QALYs) due to floor effects found when using the SF-6D and ceiling effects with the EQ-5D in different study populations [79]. In addition, although both the SF-36 and EQ-5D appear to respond to changes in depression, the agreement between utility changes is low [80]. These procedures will inform the economic evaluation plan for the design of a future phase III RCT.

### Qualitative

The five-stage framework approach [81] will be used to analyze the verbatim notes and transcribed digital recordings from the interviews. Trustworthiness of the analysis will be established by the use of triangulation by observation, whereby completed analyses conducted by JW will be sent to one other researcher and a member of the lay steering committee to discuss whether the analysis reflects the generated themes [82]. Once the second analysis is complete participants will be sent a summary of the findings to confirm whether the analysis represents accurately their experiences of the intervention [82].

### Ethical approval

We will conduct the trial in accordance with the Helsinki Declaration to safeguard the welfare and rights of participants. Ethical approval was received by the National Research Ethics Committee South West for Cornwall and Plymouth on 24 May 2013. REC Reference number: 13/SW/0018. The Data Protection Act will be followed at all times with all data securely stored and anonymized.

## Discussion

This feasibility RCT has been designed to explore important feasibility questions that can be used to inform the design and funding application of a possible future definitive (Phase III) RCT. Furthermore, detailed exploration of the acceptability of the new CBTsh intervention will inform future treatment iterations.

A supported CBTsh intervention, tailored to the needs of informal carers of stroke survivors, may represent an effective and accessible psychological intervention for depression. As well as improving mood, supported CBTsh may also improve informal carers’ quality of life and reduce carer strain and burden. Furthermore, improvements in carer depression may also improve recovery outcomes in stroke survivors themselves and represent a cost-effective model of care both nationally and internationally.

### Trial status

Recruitment commenced in September 2013 and is ongoing.

## Abbreviations

BI: Barthel activities of daily living index; CBS: Caregiver burden scale; CBTsh: Cognitive-behavioral therapy self-help; CIS-R: Clinical interview schedule; CONSORT: Consolidated standards of reporting trials; CSRI: Client socio-demographic and service receipt inventory; EQ-5D: EuroQol-5D; FAI: Frenchay activities index; GAD-7: Generalized anxiety disorder 7-item scale; GP: General practice; IAPT: Improving access to psychological therapies program; ICD-10: International classification of diseases-10; MRC: Medical research council; NHS: National health service; PHQ-9: Patient health questionnaire-9; PTSD: Post-traumatic stress disorder; PWP: Psychological wellbeing practitioner; QALY: Quality-adjusted life-year; RCT: Randomized controlled trial; SF-36: Short form (36) health survey; SF-6D: The short form-6 dimension; TAU: Treatment-as-usual; WSAS: Work and social adjustment scale.

## Competing interests

The authors declare they have no competing interests.

## Authors’ contributions

JW: conception, design, data collection, manuscript writing and final approval of the manuscript. PF: conception, design, manuscript writing and final approval of the manuscript. EW: critical revision and final approval of the manuscript. DR: critical revision and final approval of the manuscript. DL: critical revision and final approval of the manuscript. All authors have approved the final manuscript.
